# Assessing variants of uncertain significance implicated in hearing loss using a comprehensive deafness proteome

**DOI:** 10.1007/s00439-023-02559-9

**Published:** 2023-04-22

**Authors:** Mallory R. Tollefson, Rose A. Gogal, A. Monique Weaver, Amanda M. Schaefer, Robert J. Marini, Hela Azaiez, Diana L. Kolbe, Donghong Wang, Amy E. Weaver, Thomas L. Casavant, Terry A. Braun, Richard J. H. Smith, Michael J. Schnieders

**Affiliations:** 1 Roy J. Carver Department of Biomedical Engineering, University of Iowa, Iowa City, IA 52242 USA; 2 Molecular Otolaryngology and Renal Research Laboratories, Department of Otolaryngology, University of Iowa Hospitals and Clinics, Iowa City, IA 52242 USA; 3 Department of Biochemistry and Molecular Biology, University of Iowa, Iowa City, IA 52242 USA

## Abstract

Hearing loss is the leading sensory deficit, affecting ~ 5% of the population. It exhibits remarkable heterogeneity across 223 genes with 6328 pathogenic missense variants, making deafness-specific expertise a prerequisite for ascribing phenotypic consequences to genetic variants. Deafness-implicated variants are curated in the Deafness Variation Database (DVD) after classification by a genetic hearing loss expert panel and thorough informatics pipeline. However, seventy percent of the 128,167 missense variants in the DVD are “variants of uncertain significance” (VUS) due to insufficient evidence for classification. Here, we use the deep learning protein prediction algorithm, AlphaFold2, to curate structures for all DVD genes. We refine these structures with global optimization and the AMOEBA force field and use DDGun3D to predict folding free energy differences (∆∆G_Fold_) for all DVD missense variants. We find that 5772 VUSs have a large, destabilizing ∆∆G_Fold_ that is consistent with pathogenic variants. When also filtered for CADD scores (> 25.7), we determine 3456 VUSs are likely pathogenic at a probability of 99.0%. Of the 224 genes in the DVD, 166 genes (74%) exhibit one or more missense variants predicted to cause a pathogenic change in protein folding stability. The VUSs prioritized here affect 119 patients (~ 3% of cases) sequenced by the OtoSCOPE targeted panel. Approximately half of these patients previously received an inconclusive report, and reclassification of these VUSs as pathogenic provides a new genetic diagnosis for six patients.

**Supplementary Information:**

The online version contains supplementary material available at 10.1007/s00439-023-02559-9.

## Introduction

Hearing loss is the most prevalent sensory deficit, affecting approximately 5% of the world’s population (Smith et al. 2005). In its evaluation, following an audiogram, genetic sequencing with a multi-gene panel is recommended as the most informative diagnostic test for infants and children with hearing loss (Alford et al. 2014; Li et al. 2022; Liming et al. 2016; Shearer and Smith 2015). It facilitates identification of an underlying cause in 40–56% of patients in an outbred population (Shearer and Smith 2015) and up to 72% in certain ethnicities (Sloan-Heggen et al. 2016). Currently, most panel-based tests screen 23–245 genes for variants that may be implicated in hearing loss (Sloan-Heggen and Smith 2016). OtoSCOPE (Table 1), the panel we first developed in 2010 (Shearer et al. 2010), contains 223 genes in its current iteration (version 9), which in aggregate includes approximately 592,770 nucleotides of coding sequence.

**Table 1:** Abbreviations for tools, software, and computational resources used in this work

Term	References	Explanation
OtoSCOPE	(Shearer et al. 2010)	A deafness-specific genetic sequencing panel that targets 223 genes as of version 9
DVD	(Azaiez et al. 2018)	The Deafness Variation Database, a comprehensive collection of variants for the genes sequenced by the OtoSCOPE platform
AlphaFold2	(Jumper et al. 2021; Tunyasuvunakool et al. 2021)	An artificial intelligence program developed by DeepMind that predicts a protein structure starting from its amino acid sequence
FFX	(Schnieders 2021)	Force Field X, a software package for biophysical molecular modeling
OtoProtein2	(Tollefson et al. 2019)	A novel dataset of deafness-specific protein structures developed through (1) prediction by AlphaFold2 and (2) rigorous optimization with the biophysics software package, Force Field X
DDGun3D	(Montanucci et al. 2022, 2019)	A computational method for predicting the change in protein stability induced by missense variants
∆∆G_Fold_		A folding free energy difference, which quantifies the degree of protein misfolding caused by a variant
∆∆G_Bind_		A binding free energy difference, which quantifies the degree of protein binding disruption caused by a variant
CADD	(Rentzsch et al. 2018)	A widely used computational tool for predicting variant deleteriousness

In each patient screened, an average of 545 genetic variants is identified (Shearer et al. 2013). Ascribing a pathogenic consequence to these variants is challenging and requires deafness-specific expertise. To help meet this challenge, we developed the Deafness Variation Database (Azaiez et al. 2018) (DVD). The DVD includes 128,167 missense variants, which are classified by a genetic hearing loss expert panel and thorough informatics pipeline into one of five categories: benign (B, n = 1725), likely benign (LB, n = 27,907), likely pathogenic (LP, n = 2441), pathogenic (P, n = 6328), and variant of uncertain significance (VUS, n = 89,766). If a variant is classified as a VUS, a definitive diagnosis cannot be made for patients affected by that variant. For variant reclassification, additional studies are required and can include family segregation analysis, identification of the variant in a family member with hearing loss or an unrelated proband, or specific wet lab based functional evidence (Richards et al. 2015). Given the disproportionate number of VUSs, making genotype–phenotype correlations from such evidence is infeasible. Therefore, we sought to apply deep learning-based protein structure prediction (Jumper et al. 2021), atomic resolution simulation (Tollefson et al. 2019), and thermodynamic analysis (Montanucci et al. 2019, 2022) to all DVD missense variants classified as VUSs to determine whether it would be possible to reclassify some VUSs as P.

In 2019, protein structures of deafness-associated genes were known for fewer than 40% of all proteins and missense variants implicated in hearing loss (Tollefson et al. 2019), relegating computational structural variant analysis to only those variants with solved protein structures. The release of AlphaFold2 (Jumper et al. 2021; Tunyasuvunakool et al. 2021), a neural network for prediction of protein structures from sequence alone, enabled prediction of proteins with an accuracy comparable to experimentally obtained structures (Bouatta and AlQuraishi 2023). Using AlphaFold2, creation of a comprehensive deafness proteome followed by computational structural analysis of all deafness-associated variants became possible.

It is well recognized that a protein’s function and its stability are related (Araya et al. 2012; Talley and Alexov 2010). On that basis, computational folding free energy differences (∆∆G_Fold_), which quantify the change in protein folding stability caused by a variant based on thermodynamics principles (see supplementary information), have been used to characterize genes and missense variants implicated in deafness (Buonfiglio et al. 2022) including protein-specific studies (*e.g. FGFR1* (Doss et al. 2012), *TMC1* (Hilgert et al. 2008), *PNPT1* (Bereshneh et al. 2021), *PRPS1* (Agrahari et al. 2018)). When a missense variant results in protein misfolding, the protein may be targeted for degradation (Balchin et al. 2016; Goldberg 2003; McCafferty and Sergeev 2016; Stein et al. 2019). With AlphaFold2 protein structures, ∆∆G_Fold_ analysis and an accompanying prediction of protein misfolding, abrogated function and possible degradation can be done on a deafness proteome wide basis. However, computing ∆∆G_Fold_ using AlphaFold2 predicted protein structures and rigorous molecular dynamics-based simulation for all 128,167 missense variants listed in the DVD is currently intractable due to computational expense.

As an alternative, we use the high-throughput computational tool, DDGun3D (Montanucci et al. 2019), to predict ∆∆G_Fold_ (Guerois et al. 2002; Montanucci et al. 2019; Parthiban et al. 2006; Rodrigues et al. 2021; Zhou and Zhou 2002) and identify VUSs most likely to induce significant protein misfolding (often ∆∆G_Fold_ > 2–3 kcal/mol), potentially allowing these variants to be classified as P. First, we use AlphaFold2 to curate full-length, isoform-specific protein structures for all DVD genes into a protein structural database called OtoProtein2. We then reduce biophysical inaccuracies (*i.e.*, steric clashes and side-chain errors) in the OtoProtein2 structures by optimizing them with the Force Field X (FFX) biomolecular software package, which includes a global side-chain optimization algorithm (Tollefson et al. 2019) that utilizes the AMOEBA (Ponder et al. 2010) polarizable force field. Finally, we use DDGun3D (Montanucci et al. 2019) to predict ∆∆G_Fold_ for all missense variants in the DVD and resolve classifications for VUSs that cause significant changes to protein stability.

We find that 5772 VUSs have a ∆∆G_Fold_ consistent with P variants. When filtered for high CADD (Rentzsch et al. 2018) scores (> 25.7) in addition to large ∆∆G_Fold_, we identify 3456 destabilizing VUSs that are P at a probability of 99.0%. Of the 224 genes in the DVD, 166 genes (74%) have one or more missense variants predicted to cause a pathogenic change in protein folding stability. These priority VUSs affect 119 patients sequenced by OtoSCOPE (~ 3% of cases), half of whom previously received inconclusive reports. Finally, an upgraded classification of P for these priority VUSs results in a definitive genetic diagnosis for six patients.

## Materials and methods

### Predicting deafness protein structures with deep learning

We used the AlphaFold2 (Jumper et al. 2021) deep learning algorithm to predict isoform-specific protein structures for the 218 protein-coding genes in the Deafness Variation Database (Azaiez et al. 2018) (DVD). Trained on experimentally known protein structures from the Protein Data Bank (PDB) (Berman et al. 2000), the AlphaFold2 neural network predicts protein structures from amino acid sequences to an accuracy comparable to experimental results using two modules (Jumper et al. 2021). The first module develops a general hypothesis for the protein’s structure from relationships between co-evolving amino acids associated with a multiple sequence alignment. The second module predicts the spatial relationships between subsequent amino acids to produce an explicit three-dimensional protein structure. By default, the two modules are generally applied in three iterative cycles to refine the structure prediction; however, based on prior work (Mirdita et al. 2022) we applied the modules in 15 cycles to achieve higher quality predictions.

### Biophysical refinement of the AlphaFold2 deafness proteome

To improve the biophysics of the AlphaFold2 protein predictions (*i.e.*, reduce atomic clashes, choose favorable amino acid side-chain conformations, etc.), we employed both local and global optimization techniques with the AMOEBA (Ponder et al. 2010; Shi et al. 2013) polarizable force field. We first locally minimized all AlphaFold2 protein structures with the limited memory Broyden-Fletcher-Goldfarb-Shanno quasi-Newton minimization to relax the backbone and reduce atomic clashes in each protein. After local minimization, we applied a global amino acid side-chain optimization algorithm (Tollefson et al. 2019) to determine energetically favorable side-chain conformations for the amino acids in the AlphaFold2 proteins. We then used the heuristic MolProbity (Chen et al. 2010; Davis et al. 2007) algorithm to evaluate structures before and after optimization to quantify for each protein the improvement in atomic clashes, backbone angles, and side-chain conformations.

### Predicting ∆∆G_fold_ and prioritizing missense variants in the DVD

We predicted ∆∆G_Fold_ for every missense variant in the DVD (Azaiez et al. 2018) using the optimized protein structures and the high throughput computational method DDGun3D (Montanucci et al. 2019). DDGun3D (Montanucci et al. 2019) predicts a ∆∆G_Fold_ by assessing the biochemical features of a variant using its three-dimensional protein structure. We compared the distribution of ∆∆G_Fold_ in variants with P and B DVD (Azaiez et al. 2018) classifications. DVD variants are standardized on the GRCh37 reference human genome, so we used the Lift Genome Annotation (Kent et al. 2002) tool to include GRCh38 equivalent coordinates in our analysis. Using thermodynamic (see supplementary information) observations, we identified a ∆∆G_Fold_ threshold to predict genetic variants that induce significant misfolding, loss of function and possibly protein degradation. We used classified DVD variants to determine the positive predictive value (PPV) of this ∆∆G_Fold_ threshold (see confusion matrix in supplementary information). We applied this threshold to all P variants to determine which P variants are deleterious due to protein misfolding. We further applied this threshold to all VUSs in the DVD to determine which VUSs most likely impact protein misfolding and are therefore most likely to be P.

### Integrating CADD scores with ∆∆G_Fold_ to prioritize variants

We combined the ∆∆G_Fold_ predictions and threshold with CADD (Rentzsch et al. 2018) scores to prioritize VUSs most likely to be deleterious. Because variants with higher CADD scores are predicted to be more damaging (Rentzsch et al. 2018), we anticipated variants with both a large ∆∆G_Fold_ and a high CADD score are more likely to be P. We set the CADD score threshold (25.7) to reflect a 99% PPV for classified DVD variants to be P when both ∆∆G_Fold_ and CADD scores are combined. We then applied both the CADD threshold and the ∆∆G_Fold_ threshold to identify VUSs that are deleterious with 99% certainty.

### Curating variant features for further analysis

In addition to annotating ∆∆G_Fold_ and CADD scores for each DVD variant, we aggregated features from the optimized structures to be used for variant analysis, prioritization, and deep learning. For each variant, we collected AlphaFold2’s confidence in the protein structure at that variant’s position, which can be used to prioritize analysis of variants in regions where protein structure is predicted with a high degree of confidence. Similarly, because amino acids buried within a protein domain are often intolerant of variation as compared to amino acids on the surface of a protein domain, we computed the percent of solvent accessible surface area (SASA) for each DVD variant. Finally, previous work has shown that minor allele frequency (MAF) can be used to classify common variants as LB in deafness-associated genes (Shearer et al. 2014); therefore, we included the MAF for each variant in the dataset of variant features.

## Results

### Quality and characteristics of deafness protein structure predictions

**Fig. 1:**
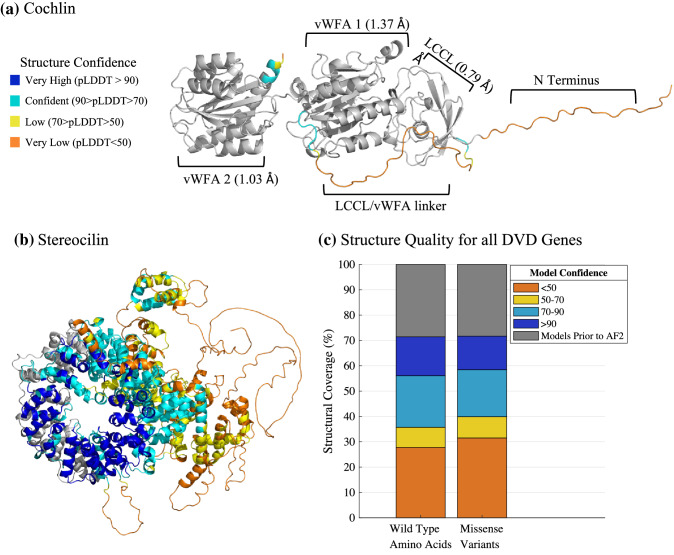
Structures and quality of proteins implicated in deafness. AlphaFold2’s novel predicted protein regions are color coded by confidence in the prediction. Gray domains represent homology or experimental structures curated in prior work for **a** cochlin and **b** stereocilin. **a** The root-mean-square deviation (RMSD) of the LCCL and vWFA domains of cochlin (*COCH*) from AlphaFold2’s domain predictions to the previous models are shown in parentheses. **b** AlphaFold2 increased protein structural coverage of stereocilin (*STRC*) from 12 to 100%. However, our work improved the quality of sterocilin’s structure through optimization (described in Methods). As a result of our optimization, the MolProbity score (described in Materials and methods) of the *STRC* structure improved from 3.07 to 0.98. **c** Structural model coverage of wild-type amino acids and missense variants for the entire deafness proteome shows that this work increased coverage from < 30% (gray, prior work) to 100% coverage. The stacked bars are color coded based on confidence in the protein structure. The wild-type amino acids and missense variants in the deafness proteome are present in similar proportions across all structural confidence ranges, indicating that specific confidence regions are not enriched for the presence of missense variants

Using AlphaFold2, we developed complete protein structures for all genes and relevant isoforms in the Deafness Variation Database (Azaiez et al. 2018) (DVD, Fig. 1a, b). Called OtoProtein2, this dataset increases structural coverage of the deafness proteome from approximately 30% by experimental and homology protein structures curated during prior work (Tollefson et al. 2019) (*i.e.*, called OtoProtein) to 100% (Fig. 1c, Figure S1). For each amino acid in a prediction, AlphaFold2 provides a unitless confidence score ranging from 1 to 100, with higher scores corresponding to higher confidence in the prediction. Model confidence is > 70 for 64% of wild-type amino acids and 60% of missense variant locations in the deafness proteome. The remaining amino acids and missense variants fall in regions that are predicted only with low confidence (*i.e.*, confidence < 70).

Approximately 41% of missense variants in the deafness proteome fall within protein regions that have an InterPro (Apweiler et al. 2001; Blum et al. 2021) domain annotation. Around 59% of variants belong to protein regions without an InterPro domain annotation (Table 2, Table S1), and these residues likely consist of flexible termini, natively disordered protein whose function has not been determined, or undiscovered protein domains. The high confidence regions of our protein predictions are enriched with InterPro domain annotations (*i.e.,* including many functional and structural domains), while the lower confidence regions of our protein predictions have few InterPro annotations and are primarily disordered. Of the 128,167 missense variants in the deafness proteome, 34% fall within the overlap of annotated InterPro domains and high confidence structural regions. Although missense variants are evenly distributed across InterPro annotated domains (*e.g.*, 41.3% and 41.4% of wild-type amino acids and missense variants are in an annotated domain, respectively), B and LB variants favor the lower confidence, unannotated regions while P and LP variants favor higher confidence regions with domain annotations (Table 3, Table S2). However, up to a quarter of P and LP variants remain in lower confidence predictions with disordered protein. Because these variants are in disordered protein, their effect on protein folding is minimal (see ∆∆G_Fold_ results). This observation indicates these variants do not cause protein misfolding and are therefore pathogenic for a different reason (*i.e.*, protein binding disruption, interruption of a post-translational modification site, etc*.*). For example, some flexible regions of protein are known to fold in the presence of a binding partner (Fuxreiter 2020), meaning that these P and LP variants may be pathogenic due to the disruption of protein binding (see discussion for more detail on binding free energy differences).

**Table 2:** Number and percent of Deafness Variation Database missense variants belonging to each AlphaFold2 confidence range based on domain annotations by InterPro

	Model confidence			
Annotated by InterPro	< 50	50–70	70–90	> 90
Yes (41.3%)	3371 (2.6%)	5610 (4.4%)	23,991 (18.7%)	20,028 (15.6%)
No (58.6%)	40,230 (31.4%)	8753 (6.8%)	15,505 (12.1%)	10,679 (8.3%)
Total	43,611 (34.0%)	14,393 (11.2%)	39,574 (30.8%)	30,956 (23.9%)

**Table 3:** Number and percent of Deafness Variation Database missense variants belonging to each AlphaFold2 confidence range based on Deafness Variation Database classification

	Model confidence			
DVD classification	< 50	50–70	70–90	> 90
B (1.4%)	719 (0.6%)	231 (0.2%)	506 (0.4%)	269 (0.2%)
LB (21.8%)	15,395 (12.0%)	3153 (2.5%)	5973 (4.7%)	3386 (2.6%)
LP (2.1%)	579 (0.5%)	203 (0.2%)	827 (0.7%)	832 (0.7%)
P (4.9%)	1201 (0.9%)	470 (0.4%)	2084 (1.6%)	2573 (2.0%)
VUS (70.1%)	25,707 (20.1%)	10,306 (8.0%)	30,106 (23.5%)	23,647 (18.5%)
Total	43,611 (34.1%)	14,393 (11.3%)	39,574 (30.9%)	30,956 (24.0%)

### Biophysical refinement of the protein structure predictions

**Fig. 2:**
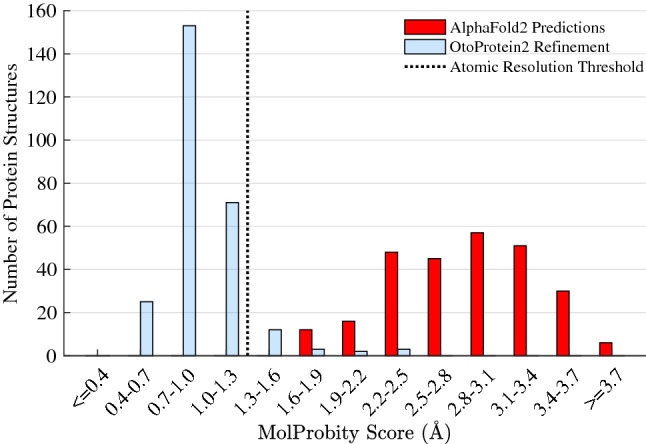
MolProbity score histogram for the OtoProtein2 database. Before optimization (red), the mean MolProbity score of the models is 2.86 and after optimization (blue) the structures are consistent with atomic resolution at a mean MolProbity score of 0.97. MolProbity scores are calibrated to reflect the expected crystallographic resolution of the diffraction dataset employed to create a protein structural model (*i.e.*, a MolProbity score of 1.0 indicates that the structure is consistent with 1.0 Å resolution X-ray diffraction data)

We applied a global side-chain optimization algorithm (Tollefson et al. 2019) and local minimization with the AMOEBA force field to each of the OtoProtein2 structures, assessing the quality of the structures before and after optimization using the MolProbity algorithm. Compared to the initial deep learning predictions from AlphaFold2, the optimization protocol reduced the average number of implausible overlaps between non-bonded atoms in each protein model (*i.e.*, steric clashes) from 20.75 to 0.11 per 1000 atoms, lowered the percent of side-chain atoms in unfavorable, high-energy conformations (*i.e.*, poor rotamers) from 4.32% to 1.12%, decreased the percent of backbone atoms that are in unfavorable dihedral angles (*i.e.*, backbone outliers) from 15.25% to 1.05%, and increased the percent of backbone atoms in favorable angles (*i.e.*, favored backbones) from 76.21 to 93.50% (Table 4). Overall, the optimization procedure improved the dataset’s mean MolProbity score from 2.86 to 0.97 (Fig. 2), making the OtoProtein2 structural quality equivalent to experimental structures at atomic resolution.

**Table 4:** Average MolProbity refinement statistics for all deafness associated protein models in OtoProtein2 before and after optimization with Force Field X

Optimization	Clash score	Poor rotamers (%)	Favored backbones (%)	Backbone outliers (%)	MolProbity Score
AlphaFold2	20.75	4.32	76.21	15.25	2.86
OtoProtein2	0.11	1.12	93.50	1.05	0.97

A lower clash score, a lower percentage of poor rotamers, a higher percentage of favored backbone phi/psi angles, fewer backbone outliers and lower MolProbity score are each better

We have incorporated the optimized OtoProtein2 structures with the DVD (www.deafnessvariationdatabase.org) to be visualized in the context of the comprehensive genetic information available therein. With 100% coverage, any DVD missense variant can be selected and visualized on its corresponding protein structure. These structures are also available for download on Github (https://github.com/SchniedersLab/OtoProtein).

### Using ∆∆G_Fold_ predictions to prioritize variants of uncertain significance

**Fig. 3:**
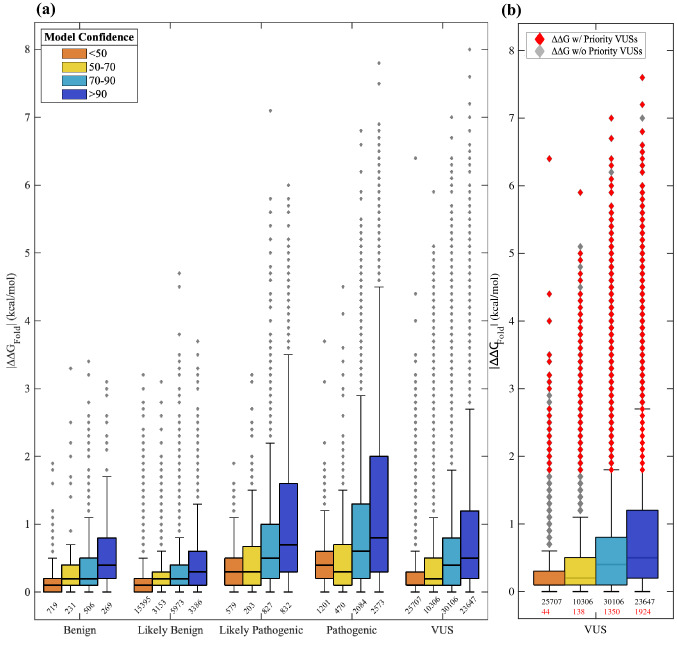
The range of ∆∆G_Fold_ predictions for missense variants in the Deafness Variation Database (DVD). **a** Box plots are grouped based on DVD pathogenicity classification and bars are colored based on the structure confidence at the variant’s amino acid position. Pathogenic variants and variants in confident portions of protein models have a larger distribution of ∆∆G_Fold_ than the benign and low confidence (*e.g.*, usually solvent exposed) counterparts. The number of observations belonging to each box is printed below the box, and each outlier in the boxplot can represent multiple VUSs due to overlap in ∆∆G_Fold_ (also applies to panel b). **b** A box plot for all VUSs in the DVD. Outliers colored in red are prioritized VUSs that have a large ∆∆G_Fold_ (≥ 1.8 kcal/mol) and a high CADD score (> 25.7). Unprioritized VUSs do not have a high CADD score. The number of prioritized VUSs belonging to each box is printed in red below the total number of observations belonging to the box

We used DDGun3D (Montanucci et al. 2019) and the optimized OtoProtein2 structures to predict the folding free energy differences (∆∆G_Fold_) for 128,167 missense variants in the DVD (Fig. 3 and Table S4). In total, 75,072 variants (59%) are destabilizing (∆∆G_Fold_ > 0), 34,253 variants (27%) are stabilizing (∆∆G_Fold_ < 0), and the remainder are neutral (∆∆G_Fold_ = 0). B variants show a mildly destabilizing mean ∆∆G_Fold_ of 0.13 kcal/mol while P variants have a higher destabilizing mean ∆∆G_Fold_ of 0.80 kcal/mol (p-value = 8.54 × 10^–197^). Within each variant classification (B: p-value = 1.006 × 10^–2^; P: p-value = 3.68 × 10^–114^), variants in high confidence regions of a protein structure (*i.e.*, often functional regions) have a higher mean ∆∆G_Fold_ and a wider distribution of ∆∆G_Fold_ than variants that fall within low confidence regions (*i.e.*, often natively disordered protein regions).

Using thermodynamics principles (see derivation in supplementary information and Table S3), a ∆∆G_Fold_ of > 1.8 kcal/mol represents a 20-fold decrease in the ratio of folded to unfolded protein. At this threshold, variants with a ∆∆G_Fold_ larger than 1.8 kcal/mol are appreciably destabilizing to a protein fold, likely resulting in loss of function or protein degradation. The 1.8 kcal/mol threshold results in a positive predictive value (PPV) of 97.1% and specificity of 98.2%, with nearly 17% of pathogenic variants (1067 of all P variants in the DVD) falling above 1.8 kcal/mol. Using the ∆∆G_Fold_ with a 1.8 kcal/mol cutoff, 5772 VUSs are deleterious due to destabilization of the protein fold, loss of native function and possibly protein degradation. The presence of both destabilizing and over stabilizing variants are known to result in disease phenotypes (Stefl et al. 2013; Takano et al. 2012; Witham et al. 2011), and we observed that some pathogenic DVD variants have a largely over stabilizing ∆∆G_Fold_ (< − 1.8 kcal/mol). However, using a − 1.8 kcal/mol threshold (*i.e.*, a 20-fold increase in the ratio of folded to unfolded protein) to identify over stabilizing variants resulted in a PPV of only 93.0% and applied to only 53 pathogenic variants. Therefore, we focused attention on only destabilizing variants. With nearly 90,000 VUSs in the DVD, DDGun3D provides an efficient means for calculating ∆∆G_Fold_ and identifying deleterious variants.

### Integrating CADD scores with ∆∆G_Fold_ to prioritize VUSs

**Fig. 4:**
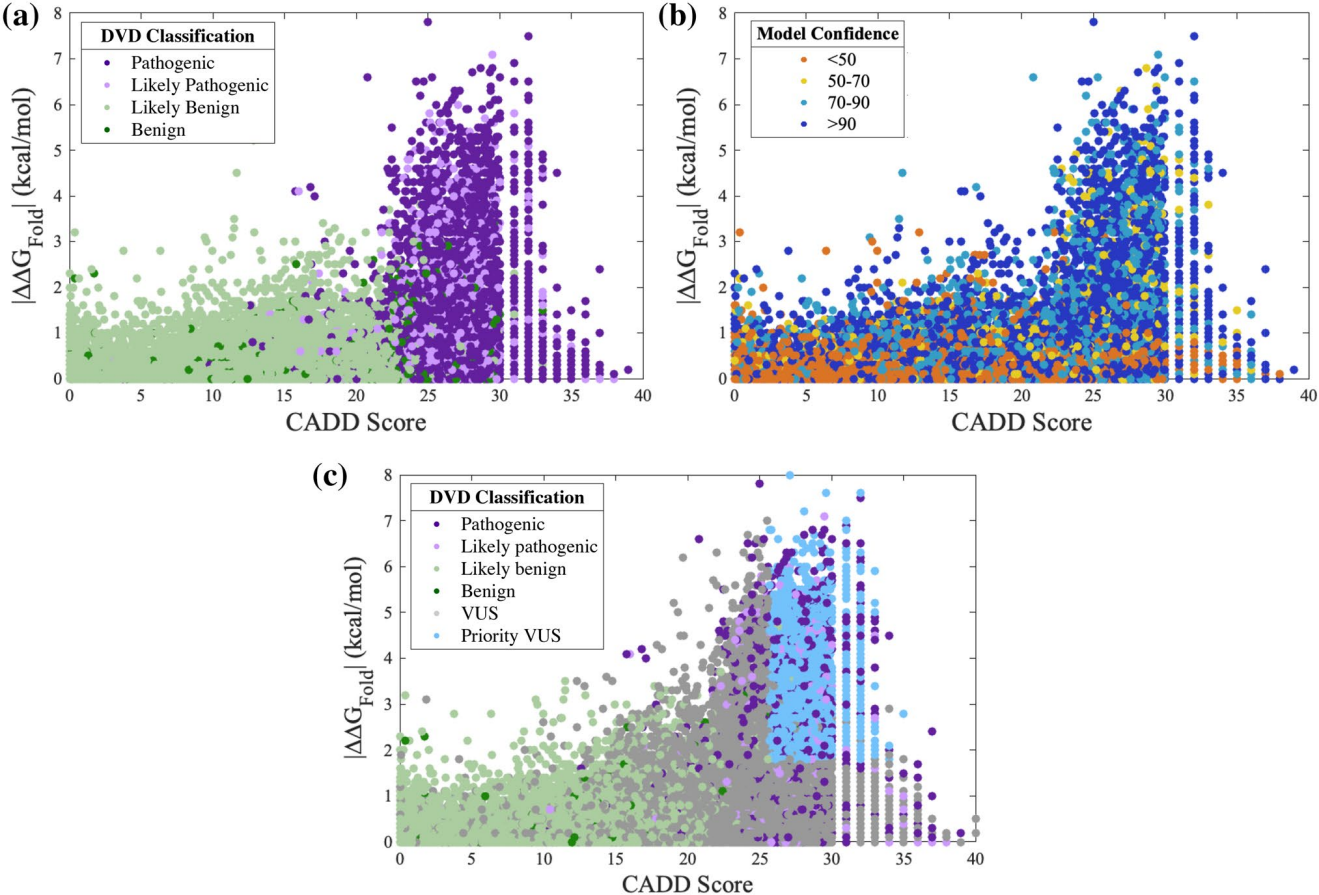
Prioritizing variants of uncertain significance (VUSs) from folding free energy differences (∆∆G_Fold_) and CADD scores. The ∆∆G_Fold_ versus CADD score for all classified missense variants (panels **a** and **b**) and for all variants including VUSs (panel **c**) observed in the Deafness Variation Database (DVD). Points are colored according to DVD classification (panels **a** and **c**) or model confidence at the variant’s amino acid position (panel **b**). CADD score and ∆∆G_Fold_ show a positive correlation. A high ∆∆G_Fold_ and high CADD score in confident regions of a protein model favor pathogenic variants; low ∆∆G_Fold_ and low CADD score favor benign variants and exhibit greater variety in model confidence. Prioritized VUSs have both high ∆∆G_Fold_ and high CADD scores

CADD scores (Rentzsch et al. 2018) can be used in combination with ∆∆G_Fold_ to prioritize variants most likely to be deleterious. Higher CADD scores are associated with P and LP variants (Fig. 4a, Figure S2). These variants also favor protein regions with high confidence (Fig. 4b) and consist primarily of domains and motifs that are intolerant to variation. Establishing a CADD threshold independently has a reasonable PPV (*e.g.*, a CADD cutoff of 20 results in a PPV of 88.3%). We applied a CADD cutoff of 25.7 and combined this threshold with the ∆∆G_Fold_ threshold, which resulted in a PPV of 99% and a specificity of 99.5%. While these stringent CADD and ∆∆G_Fold_ thresholds limit prioritization to 3456 destabilizing VUSs (Table 5 and Table S5), these VUSs can be classified as LP due to protein misfolding (Fig. 4c, Figure S3). In total, of the 224 genes in the DVD, 166 genes are affected by the 3456 prioritized VUSs and are therefore, susceptible to protein misfolding.

**Table 5:** Summary of genes with 30 or more prioritized VUSs per 1000 amino acids in length (*i.e.*, variant density)

Gene	Protein family	Variant density	Protein length	# VUSs	Mean ∆∆G_Fold_	Mean CADD
*ATP6V1B1*	ATPase	33.1	513	17	2.8	27.8
*CDC14A*	Tyrosine phosphatase	36.9	623	23	2.7	28.3
*CLRN1*	Clarin	43.1	232	10	2.3	26.6
*DCAF17*	Not assigned	42.3	520	22	3.2	27.9
*DIABLO*	Not assigned	58.6	239	14	2.7	28.4
*ELMOD3*	Not assigned	31.5	381	12	3.1	27.6
*GIPC3*	GIPC	41.7	312	13	3.1	27.5
*GJB2*	Connexin	44.2	226	10	3.1	27.6
*GJB3*	Connexin	40.7	270	11	3.4	27.3
*GRXCR1*	GRXCR1	34.5	290	10	2.8	27.6
*GSDME*	Gasdermin	38.3	496	19	3.4	27.4
*HARS2*	Aminoacyl-tRNA synthetase	33.2	512	17	2.7	28.8
*KARS1*	Aminoacyl-tRNA synthetase	30.4	625	19	2.7	28.3
*LHFPL5*	LHFP	32.0	219	7	3.3	28.4
*LOXL3*	Lysyl oxidase	35.9	753	27	3.0	27.8
*MANBA*	Glycosyl hydrolase	30.7	879	27	3.1	27.7
*MASP1*	Peptidase	50.8	728	37	3.1	28.6
*MSRB3*	Sulfoxide reductase	37.8	185	7	3.4	28.3
*MYO3A*	Myosin-kinesin ATPase	38.4	1616	62	3.0	28.4
*MYO6*	Myosin-kinesin ATPase	30.9	1294	40	3.1	27.9
*MYO7A*	Myosin-kinesin ATPase	39.3	2215	87	3.0	28.0
*NARS2*	Aminoacyl-tRNA synthetase	46.1	477	22	2.8	28.3
*OTOF*	Ferlin	30.0	1997	60	3.2	28.4
*OTOGL*	Otogelin	61.0	2344	143	3.1	28.2
*PCDH15*	Not assigned	30.7	1790	55	3.1	27.9
*POLR1C*	RNA polymerase	46.2	346	16	2.9	27.9
*RDX*	Not assigned	39.7	604	24	3.0	27.5
*SEMA3E*	Semaphorin	31.0	775	24	3.3	28.1
*SLC17A8*	Sodium/anion cotransporter	30.6	589	18	2.8	28.9
*SLC19A2*	Thiamine transporter	70.4	497	35	3.5	28.1
*SLC22A4*	Cation transporter	38.1	551	21	3.0	28.3
*SLC26A4*	SLC26A/SulP transporter	57.7	780	45	2.9	28.1
*SLC44A4*	Choline transporter-like	54.9	710	39	3.0	28.5
*SLC52A2*	Riboflavin transporter	36.0	445	16	3.2	26.9
*SLC52A3*	Riboflavin transporter	34.1	469	16	3.2	27.3
*TECTA*	Not assigned	39.0	2155	84	3.1	28.0
*TMC1*	TMC	31.6	760	24	3.3	29.0
TSPEAR*	Not assigned	31.6	601	19	3.5	28.1
*WFS1*	Not assigned	51.7	890	46	3.0	27.7

A comprehensive list of all prioritized VUSs is available in Table S4. These VUSs were prioritized based on having a ∆∆G_Fold_ > 1.8 and a CADD score > 25.7

*Indicates a disputed deafness gene (Bowles et al. 2021)

**Fig. 5:**
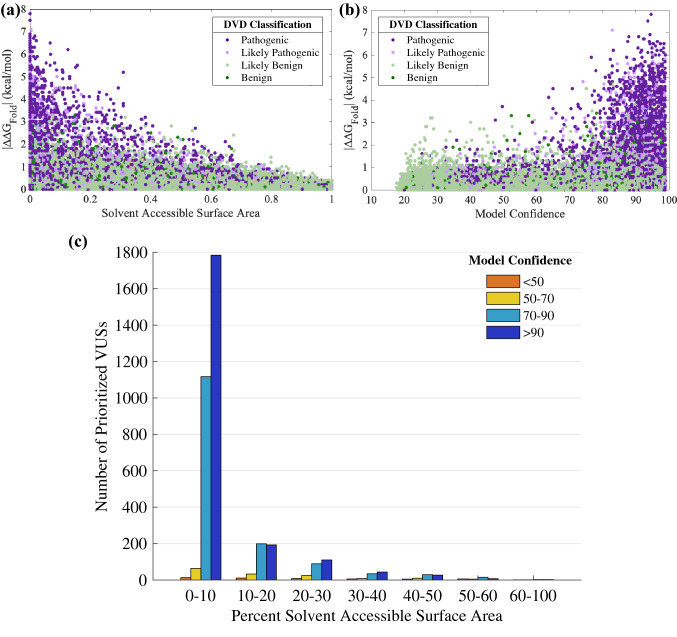
Protein features for prioritizing VUSs. Folding free energy differences (∆∆G_Fold_) versus **a** percent of solvent accessible surface area (SASA) at a variant’s amino acid position, and **b** model confidence at the variant position for all classified missense variants in the Deafness Variation Database (DVD). Pathogenic and likely pathogenic variants favor buried, high confidence protein regions. **c** A histogram of the percent SASA for all prioritized VUSs. Similar to known P and LP, the prioritized VUSs are mostly in buried, high confidence protein regions

We found that P and LP variants are often in buried residues (*i.e.*, solvent accessible surface area near zero percent) with confident structure regions (Fig. 5a, b, Figure S4, Figure S5). The prioritized dataset of 3456 VUSs are consistently present in buried, confident regions of the OtoProtein2 structures (Fig. 5c). Additionally, ∆∆G_Fold_, CADD scores, solvent accessible surface area, and structure confidence from the OtoProtein2 models for all variants in the DVD can be utilized for deep learning applications or for variant analysis.

## Discussion

The classification of genetic variation in relationship to a disease phenotype is challenging. For hearing loss, the DVD uses an expert panel and a rigorous informatics pipeline to classify changes in deafness-associated genes based on evidence of pathogenicity. This database includes over 128,167 missense variants, the majority of which (> 70%) are classified as VUSs due to insufficient evidence to classify as P or B. A VUS classification is problematic for both the healthcare provider and the patient as a definitive diagnosis cannot be made. Here we show that computationally determined ∆∆G_Fold_ can resolve a portion of VUSs by quantifying the change in protein stability induced by a variant, consequently providing insight as to the variant’s mechanism of action (*i.e.*, the variant induces protein misfolding) and its pathogenicity. We used AlphaFold2 and a global optimization algorithm (Tollefson et al. 2019) to develop OtoProtein2, a database of optimized, isoform specific, full-length protein structures for every gene in the DVD. We then used these new protein models and the computational tool, DDGun3D (Montanucci et al. 2019), to quantify the change in protein stability (*i.e.*, ∆∆G_Fold_) caused by each missense variant in the DVD. We found that ∆∆G_Fold_ greater than 1.8 kcal/mol is predictive of P variants at a rate of 97.1%. Combining large ∆∆G_Fold_ (> 1.8 kcal/mol) and large CADD scores (> 25.7) results in a positive predictive value (PPV) of 99.0%. Using these ∆∆G_Fold_ and CADD thresholds, we identified 3456 VUSs that are LP due to protein misfolding, which span 166 (74%) DVD genes. More prior work than can be efficiently summarized has led to the classification of only 2441 LP variants (and 6328 P variants) implicated in deafness, but the approaches outlined in this work increase the number of LP variants by 2.4x (from 2441 to 5897 total LP variants).

**Fig. 6:**
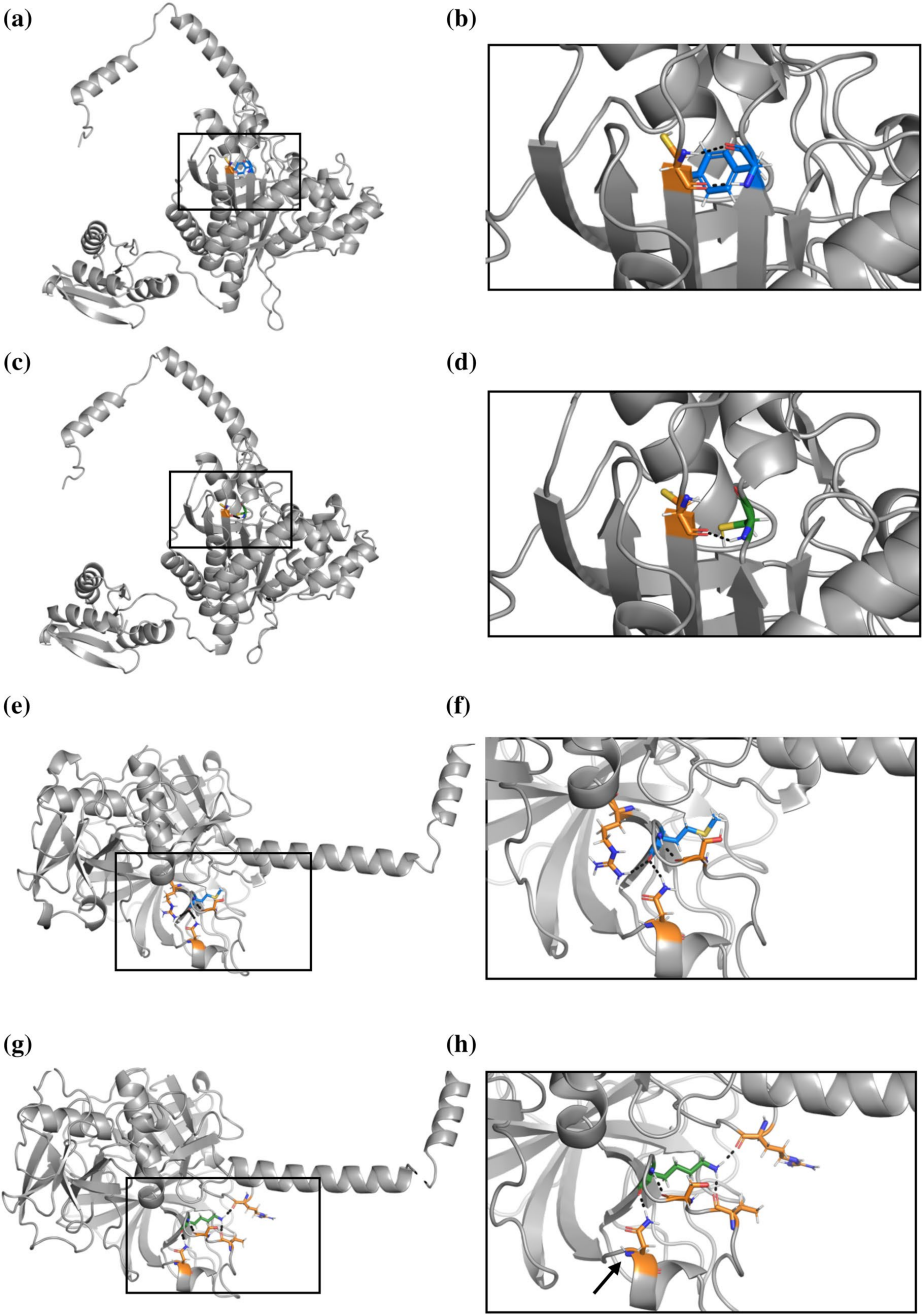
The protein structure of *HARS2* variant NP_036340.1:p.Tyr364Cys (Panels (**a**–**d**) and *TMPRSS3* variant NP_076927.1:p.Met384Lys (Panels **e–h**). All hydrogen bonds are indicated by black dashed lines. **a** The wildtype *HARS2* protein contains a tyrosine (blue) at position 364, which interacts with a neighboring cysteine amino acid (orange) **b** Augmentation of the boxed region in Panel A shows two hydrogen bonds between the tyrosine and cysteine. **c** The NP_036340.1:p.Tyr364Cys variant introduces a new cysteine (green) in place of tyrosine. **d** Enlargement of the boxed region from Panel C shows that the variant cysteine (green) interacts with the original neighboring cysteine (orange), disrupting the two hydrogen bonds to form a single hydrogen bond or a disulfide bond. **e** The wildtype *TMPRSS3* protein shows a methionine (blue) at position 384, which interacts with three neighboring amino acids (orange). **f** Magnification of Panel E shows three hydrogen bonds between the methionine and neighboring amino acids. **g** The NP_076927.1:p.Met384Lys variant introduces a lysine (green) in place of methionine, which interacts with four neighboring amino acids, only one of which remains the same as the wildtype interacting neighbors. **h** Enlargement of the boxed region from Panel G shows four hydrogen bonds between the lysine (green) and neighboring amino acids. While one hydrogen bond remains the same between the wildtype and variant structures **a** black arrow indicates the residue with the unaltered hydrogen bond), the NP_076927.1:p.Met384Lys variant results in significant misfolding

Of these 3456 prioritized VUSs, we have observed 79 across 119 patients who underwent comprehensive genetic testing using OtoSCOPE. Over half of these patients (60 patients) previously received an inconclusive genetic diagnosis. In five patients with variants affecting autosomal recessive genes, the proband carried a second LP/P variant in the gene. Segregation analysis (SA) confirmed that the second LP/P variant occurs on the opposite allele in three of five patients; in the remaining two patients, SA was not available. One patient carried a variant affecting an autosomal dominant gene. The work here delivers a definitive genetic diagnosis for these six patients and directly impacts their subsequent healthcare (Table 6). For example, patient six carried a known P variant in *TMPRSS3* in trans with a novel missense variant predicted to cause protein destabilization by this work (Fig. 6). The phenotype of the patient’s hearing loss is highly specific for *TMPRSS3*-related hearing loss (DFNB8/10). Reclassification of patient six’s novel missense variant from VUS to LP results in a definitive genetic diagnosis, ultimately directing subsequent medical care and recurrence risk calculations for offspring. Current guidelines established by the American College of Medical Genetics and Genomics (ACMG) for hearing loss do not incorporate computational ∆∆G_Fold_ calculations, however, our work demonstrates the utility of protein modeling for hearing loss diagnostics. Future work includes clinically validating the pathogenicity of Table 6 variants by following ACMG guidelines and completing familial sequencing and segregation analysis where feasible. Importantly, further work is indicated to guide incorporation of protein modeling into ACMG guidelines for hearing loss and deafness.

**Table 6:** Patients with definitive diagnoses from upgraded classification of priority VUSs

Patient ID	Gene	Inheritance	Priority VUS	Second variant (classification)	Segregation analysis
1	*CDH23*	AR	NP_071407.4:p.Tyr2883Ser	Arg2795Ter (P)	NA
2	*GRXCR1*	AR	NP_001073945.1:p.Tyr142Cys	Gln283Ter (P)	Yes
3	*HARS2*	AR	NP_036340.1:p.Tyr364Cys	Arg150Cys (LP)	Yes
4	*MYO6*	AD	NP_001355794.1:p.Cys1236Arg	None	NA
5	*PDZD7*	AR	NP_001182192.1:p.Ile269Ser	Arg56ProfsTer24 (P)	NA
6	*TMPRSS3*	AR	NP_076927.1:p.Met384Lys	His70ThrfsTer19 (P)	Yes

Segregation analysis confirms that the second variant occurs on the opposite allele in three probands, Table cells with NA are not available

The number of prioritized VUSs and impacted patients is greatly affected by adjustments to the ∆∆G_Fold_ and CADD thresholds. We used a ∆∆G_Fold_ threshold of 1.8 kcal/mol and a CADD threshold of 25.7 to reach a PPV of 99.0% (false positive rate < 0.5%), but by increasing the CADD threshold to 30.0, the PPV approaches 100%. These stringent thresholds leave negligible room for a false positive diagnosis but provide a prioritized dataset of only 419 VUSs that are LP. Seven of these 419 VUSs impact 18 OtoSCOPE patients. Alternatively, a more lenient PPV of 95% is reached by disregarding CADD scores and dropping the ∆∆G_Fold_ cutoff to 1.0 kcal/mol. These parameters provide a substantially larger dataset of 12,585 VUSs that are LP, albeit with a 5.6% false positive rate, and impact 775 OtoSCOPE patients. Prior work (Azaiez et al. 2018) has shown that approximately 1% of LP variants in deafness-implicated genes should be reclassified as LB, suggesting that existing approaches for variant classification result in an error rate close to 1% for LP variants. Although adjusting the ∆∆G_Fold_ and CADD thresholds to affect the PPV, number of prioritized VUSs that are upgraded to LP status, and number of impacted patients is possible, the thresholds resulting in a 99.0% PPV have an error rate that is in-line with existing classification techniques.

Though we applied the ∆∆G_Fold_ and CADD thresholds on a deafness-proteome-wide scale, these cutoffs can be tuned to better fit a protein, domain, or amino acid specific level. Biochemical, environmental, and structural differences contribute to a protein’s ability to tolerate changes to its structure. For example, *ACTG1* encodes gamma actin, a highly conserved cytoskeletal protein. While no P *ACTG1* variants from the DVD surpass both the ∆∆G_Fold_ and CADD cutoffs for the proteome-wide scale, it is possible even small ∆∆G_Fold_ cause enough misfolding to disrupt gamma actin’s highly conserved structure and function. Investigation of such gene specific ∆∆G_Fold_ thresholds will be the subject of future work. Similarly, different domains within an individual protein can benefit from domain-specific ∆∆G_Fold_ analysis. Cochlin, the protein product of the *COCH* gene, has one Limulus factor C (LCCL) domain and two Von Willebrand factor A (VWFA) domains. P variants in *COCH* are known to localize in the LCCL and second VWFA domains (Gallant et al. 2013). Known P variants aggregating in just one of cochlin’s two VWFA domains demonstrate the need for domain-specific analysis to identify which domains are more sensitive to amino acid variation and are intolerant of misfolding. Even individual amino acid characteristics such as the AlphaFold2 structural confidence of the wild-type amino acid, SASA, or number of hydrogen bonds can affect an amino acid’s ability to tolerate a missense variant that disrupts the protein’s structure. For example, recent work (Akdel et al. 2022) has shown that protein structures with a higher AlphaFold2 confidence show a higher concordance between experimental and predicted ∆∆G_Fold_. As approaches for ∆∆G_Fold_ predictions are improved, context-dependent thresholds will be significant for variant interpretation.

The ∆∆G_Fold_ and CADD thresholds used to identify VUSs that induce substantial protein destabilization can also provide an estimate of the number of deafness-causing genetic variants yet to be classified as P. Because ∆∆G_Fold_ quantifies the disruption to protein folding induced by variants, ∆∆G_Fold_ resolves only those VUSs that are P due to protein misfolding. Applying these thresholds to listed P and LP variants in the DVD allows us to identify that subset of missense variants that destabilize protein structure. Of the 6328 known P variants, 793 (12.5%) exceed the ∆∆G_Fold_ and CADD thresholds and fall into this category, while the remaining P variants (5535 variants, or 87.5%) are P for reasons unrelated to protein misfolding. Consequently, if the 3456 VUSs we identified as LP due to misfolding represent ~ 12.5% of the remaining deleterious variants to be found, we estimate that approximately 24,192 VUSs are P for reasons unrelated to protein misfolding.

There are two important limitations to this work: (1) the accuracy of ∆∆G_Fold_ predictions and (2) the inherent ability of ∆∆G_Fold_ to quantify only protein misfolding. With respect to the former, DDGun3D predictions of ∆∆G_Fold_ are expected to be within ~ 1.5 kcal/mol of an experimentally known ∆∆G_Fold_ (Montanucci et al. 2019), and the leading molecular dynamics software (FEP +) for calculating ∆∆G_Fold_ is within ~ 1.1 kcal/mol of the experimentally known values (Duan et al. 2020). While this degree of accuracy is sufficient to identify VUSs that are LP (*i.e.*, impact protein folding), more refinement may be needed for validating and discriminating amongst highly similar variants. There is, however, a trade-off in time. DDGun3D ∆∆G_Fold_ requires only minutes of compute time, while an equivalent ∆∆G_Fold_ calculation (Duan et al. 2020) with the Nanoscale Molecular Dynamics (NAMD) software package (Chen et al. 2020) requires on the order of one month of simulation time using a Graphical Processing Unit (GPU). This time increase also makes calculating ∆∆G_Fold_ with FEP +  (Duan et al. 2020) or NAMD too computationally expensive for a dataset of 128,167 variants. However, these simulations may be suitable for systematically improving ∆∆G_Fold_ results of the most noteworthy prioritized VUSs or for validation prior to wet-lab experiments.

With respect to the second limitation, ∆∆G_Fold_ quantifies only the change in protein stability induced by a variant, and is therefore limited to testing the hypothesis that a missense variant disrupts protein folding (Stefl et al. 2013). Although ∆∆G_Fold_ provides a biochemical hypothesis for one mechanism by which a variant can affect protein function (*i.e.*, protein misfolding), ∆∆G_Fold_ does not test for possible pathogenicity due to reasons unrelated to protein misfolding such as interrupting an active site (Zhang et al. 2010, 2011) or altering protein–protein interactions (Teng et al. 2009).

Future directions for this work include computing binding free energy differences (*i.e.*, ∆∆G_Bind_) and expanding our analysis beyond missense variants. In contrast to ∆∆G_Fold_, ∆∆G_Bind_ quantifies the difference in binding caused by a missense variant and tests the hypothesis that a variant alters a protein–protein interaction. Accurate structures of protein complexes and sufficient knowledge of interactions are a prerequisite for computing meaningful ∆∆G_Bind_, and while progress is being made in this direction (methods such as AlphaFold2-Multimer (Bryant et al. 2022), ColabFold (Mirdita et al. 2022), and AF2Complex (Gao et al. 2022) can predict protein complexes), only ~ 20% of complex predictions are considered high accuracy according to criteria established by the Critical Assessment of Predicted Interactions (Yin et al. 2022). Further, finite hardware memory combined with the memory requirements for deep learning-based protein model predictions often require that monomeric proteins be predicted in segments. This memory limitation is only exacerbated by the prediction of protein complexes where memory limits are more easily reached. Nevertheless, attaining a comprehensive model of the deafness interactome and subsequent analysis of ∆∆G_Bind_ will be the subject of future studies. The analysis of indels, non-coding variants, and other variants, are beyond the scope of our current work, however, prioritization and characterization of these variants should be considered in context with the VUSs prioritized herein. Regardless of the work remaining, the deafness proteome and ∆∆G_Fold_ analysis we present has revealed trends for P variants and provides insight on VUSs that are LP due to protein misfolding.

In summary, by using ab initio protein structure prediction, optimization, and thermodynamic analysis, with 99% confidence, we have identified 3456 VUSs that are LP in patients with hearing loss due to protein misfolding. The deafness protein structures developed here have been incorporated with the DVD to inform deafness-associated variant analysis. As atomic resolution protein structures and computational variant analysis techniques progress, continued and refined analysis of free energy differences for deafness-associated variants will inform pathogenicity classifications and lead to enhanced patient diagnoses. All data accumulated during this project are available on Github (https://github.com/SchniedersLab/OtoProtein).

## Supplementary Information

Below is the link to the electronic supplementary material.

Supplementary file1 (DOCX 2645 KB)

Supplementary file2 (XLSX 16744 KB)

Supplementary file3 (XLSX 178 KB)

## Data Availability

The datasets generated during this study are available at https://github.com/SchniedersLab/OtoProtein, OtoProtein2 models and folding free energy differences: https://github.com/SchniedersLab/OtoProtein, Force Field X software for protein model optimization: https://ffx.biochem.uiowa.edu
