# Supplementary material for: Assessing variants of uncertain significance implicated in hearing loss using a comprehensive deafness proteome
Source: Hum Genet. 2023 Apr 22;142(6):819–34. doi: 10.1007/s00439-023-02559-9 (PMC10182131; doi:10.1007/s00439-023-02559-9)
Supplement: Supplementary file 1 — Supplementary file1 (DOCX 2645 KB) [file 439_2023_2559_MOESM1_ESM.docx]

Supplementary Information For:

Assessing Variants of Uncertain Significance Implicated in Hearing Loss Using a Comprehensive Deafness Proteome

Mallory R. Tollefson,^1,2^ Rose A. Gogal,^1^ A. Monique Weaver,^2^ Amanda M. Schaefer,^2^ Robert J. Marini,^2^ Hela Azaiez,^2^ Diana L. Kolbe,^2^ Donghong Wang,^2^ Amy E. Weaver,^2^ Thomas L. Casavant,^1^ Terry A. Braun,^1^ Richard J. H. Smith,^2,*^ and Michael J. Schnieders,^1,3,*^

^1^Roy J. Carver Department of Biomedical Engineering, University of Iowa, Iowa City, IA, 52242, USA

^2^ Molecular Otolaryngology & Renal Research Laboratories, Department of Otolaryngology, University of Iowa Hospitals and Clinics, Iowa City, IA, 52242, USA

^3^Department of Biochemistry and Molecular Biology, University of Iowa, Iowa City, IA, 52242, USA

^*^Correspondence: [michael-schnieders@uiowa.edu](mailto:michael-schnieders@uiowa.edu)

richard-smith@uiowa.edu

## Predicting Protein Models >2700 Amino Acids in Length Using AlphaFold2

The AlphaFold Protein Structure Database provides predictions for proteins more than 16 amino acids and fewer than 2700 amino acids in length. Due to finite hardware memory, proteins with more than 2700 amino acids are predicted in a segmented approach; the first 1400 amino acids are predicted, the start of the sequence is moved by 200 amino acids, a new model is created, and the cycle repeats until the end of the sequence is reached. These segmented predictions result in redundant protein models where a single amino acid position can be included in as many as eight different model predictions; consequently, any computational biochemical analysis of a specific amino acid position (*e.g.*, as in the case of a missense variant) becomes challenging. To ensure a particular amino acid position is represented by a single cohesive model, we first predicted sequences of 2400 amino acids with 200 amino acids of redundant residues to the next model. We then aligned the redundant regions of the sequential models and excised one copy of duplicated residues to form a single, continuous model. This protocol was necessary for 11 nuclear genes in the DVD that have isoforms with more than 2700 amino acids.

## Confusion Matrix and Prediction Statistics

$Positive Predicitive Value=\frac{\# of True Positives}{\# of True Positives + \# of False Positives}= \frac{796}{796+8}=99.0\%$

$Specificity=\frac{\# of True Negatives}{\# of True Negatives + \# of False Positives}= \frac{1717}{1717+8}=99.5\%$

**Figure S1.** Structural model coverage of residues and missense variants for the deafness proteome as provided by AlphaFold2. Stacked bars are color coded based on confidence in the model (pLDDT). The white portion of the stacked bar shows residues and missense variants that have no coverage due to deafness-relevant isoforms that are not included in AlphaFold2’s database.

**Figure S2.** Folding free energy differences vs. CADD score for all missense variants (main text excludes VUSs) observed in the Deafness Variation Database (DVD). Points are colored based on A) the model confidence (pLDDT) at the variant’s amino acid position and B) the variant’s DVD pathogenicity classification. DDGun free energy and CADD score show positive correlation. A high free energy and high CADD score in confident regions of a protein model favors pathogenic variants; low free energy and low CADD score favor benign variants and exhibit greater variety in model confidence.

**Figure S3.** Folding free energy differences vs. CADD score for all VUSs observed in the Deafness Variation Database (DVD). Points are colored based on A) the model confidence (pLDDT) at the variant’s amino acid position and B) the variant’s classification using our DDGun and CADD score cutoffs.

A)

B)

C)

**Figure S4.** Folding free energy differences versus A) normalized solvent accessible surface (SASA) area at a variant’s amino acid position, B) model confidence at the variant position (pLDDT), and C) a variant’s minor allele frequency (MAF) for all variants (main text excludes VUSs) in the Deafness Variation Database (DVD).

A)

B)

C)

**Figure S5.** Folding free energy differences versus A) normalized solvent accessible surface (SASA) area at a variant’s amino acid position, B) model confidence at the variant position (pLDDT), and C) a variant’s minor allele frequency (MAF) for all VUSs in the Deafness Variation Database (DVD). Colors are based on classifications from DDGun3D and CADD cutoffs.

| **Table S1.** Number and percent of Deafness Variation Database missense variants belonging to each classification based on characterized or uncharacterized InterPro domain annotations. | | | | | |
| --- | --- | --- | --- | --- | --- |
|  | DVD Classification | | | | |
| InterPro Domain | B | LB | LP | P | VUS |
| Characterized (41.5%) | 619 (0.5%) | 7394 (5.8%) | 1372 (1.1%) | 3673 (2.9%) | 39942 (31.1%) |
| Uncharacterized (58.5%) | 1106 (0.9%) | 20513 (16.0%) | 1069 (0.8%) | 2655 (2.0%) | 49824 (38.9%) |
|  |  |  |  |  |  |

| **Table S2.** Classification of variants in the Deafness Variation Database. | | |
| --- | --- | --- |
| Classification | Count | Percent |
| Benign | 1725 | 1.35% |
| Likely Benign | 27907 | 21.77% |
| Likely Pathogenic | 2441 | 1.90% |
| Pathogenic | 6328 | 4.94% |
| Variant of Uncertain Significance | 89766 | 70.04% |
| Total | 128534 | 100% |

## Folding Free Energy Differences (∆∆G_Fold_) as a Ratio of Folded/Unfolded Protein

Folding free energy differences (∆∆G_Fold_) quantify the change in protein stability induced by a variant. A ∆∆G_Fold_ is defined by Equation 1, where ∆∆G_Fold_ is the folding free energy difference between a wild type and variant protein, ∆G_V_ is the free energy difference of the variant protein and ∆G_WT_ is the free energy difference of the wild type protein.

$${\Delta\Delta G}_{\mathrm{Fold}}={\Delta G}_{V}-{\Delta G}_{\mathrm{WT}}$$

Equation 1.

The free energy difference for one protein state (*i.e.*, variant or wild type) is defined by Equation 2, where k_b_ is Boltzmann’s constant, T is temperature, and ln([Folded]/[Unfolded]) is the natural log of the concentration of folded and unfolded protein.

$$\Delta G =-k_{b}T\cdot\ln\left( \frac{[Folded]}{[Unfolded]} \right)$$

Equation 2.

Combining Equations 1 and 2 is shown by Equation 3, where the folding free energy difference (∆∆G_Fold_) is represented as the difference between the free energy difference of the variant and wild type proteins.

$${\Delta\Delta G}_{\mathrm{Fold}}=-k_{b}\mathrm{Tln}\left( \frac{{[Folded]}_{V}}{{[Unfolded]}_{V}} \right)+k_{b}\mathrm{Tln}\left( \frac{{[Folded]}_{\mathrm{WT}}}{{[Unfolded]}_{\mathrm{WT}}} \right)$$

Equation 3.

Isolating the concentration of folded and unfolded variant protein results in Equation 4, which demonstrates that as ∆∆G_Fold_ increases, the ratio of folded to unfolded variant protein decreases relative to the wild type ratio.

$$\frac{\left[ \mathrm{Folded} \right]_{V}}{\left[ \mathrm{Unfolded} \right]_{V}}=\exp\left( -\frac{{\Delta\Delta G}_{\mathrm{Folding}}}{k_{B}T} \right)\cdot\frac{\left[ \mathrm{Folded} \right]_{\mathrm{WT}}}{\left[ \mathrm{Unfolded} \right]_{\mathrm{WT}}}$$

Equation 6.

At room temperature, k_b_T is approximately 0.6 kcal/mol. Table S3 shows the relative change to [Folded]/[Unfolded] ratios based on a specific ∆∆G_Fold_.

| **Table S3.** The relative change in the [Folded]/[Unfolded] protein ratio between variant and wild type protein based on the folding free energy difference (∆∆G_Fold_). | | |
| --- | --- | --- |
| ∆∆G_Fold_ | Change to [Folded]/[Unfolded] Ratio | Potential Classification  (Purple: pathogenic; Green: benign) |
| <-2.0 | >30x Increase |  |
| -1.5 to -2.0 | 12x to 30x Increase |  |
| -1.0 to -1.5 | 5x to 12x Increase |  |
| -1.0 to 1.0 | 5x Decrease to 5x Increase |  |
| 1.0 to 1.5 | 5x to 12x Decrease |  |
| 1.5 to 2.0 | 12x to 30x Decrease |  |
| >2.0 | >30x Decrease |  |
